# Thrombotic Thrombocytopenic Purpura in Interferon Beta-1a-Treated Patient Diagnosed with Relapsing-Remitting Multiple Sclerosis: A Case Report

**DOI:** 10.3390/life12010080

**Published:** 2022-01-07

**Authors:** Cristina-Florentina Plesa, Diana Maria Chitimus, Carmen Adella Sirbu, Monica Marilena Țânțu, Minerva Claudia Ghinescu, Daniela Anghel, Florentina Ionita-Radu

**Affiliations:** 1 Department of Neurology, “Dr. Carol Davila” Central Military Emergency University Hospital, 134 Calea Plevnei Str., 010242 Bucharest, Romania; cristina.plesa@prof.utm.ro (C.-F.P.); diana.chitimus@rez.umfcd.ro (D.M.C.); 2 Department of Preclinical Disciplines, Faculty of Medicine, “Titu Maiorescu” University, 031593 Bucharest, Romania; 3 Department of Medical-Surgical and Prophylactical Disciplines, Faculty of Medicine, “Titu Maiorescu” University, 031593 Bucharest, Romania; minerva.ghinescu@prof.utm.ro; 4 Department of Health Care and Therapy, Faculty of Science, Physical Education and Informatics, Universty of Piteşti,110040 Pitești, Romania; marilena.tantu@upit.ro; 5 Department of Internal Medicine, “Dr. Carol Davila” Central Military Emergency University Hospital, 134 Calea Plevnei Str., 010242 Bucharest, Romania; daniela.anghel@prof.utm.ro; 6 Department of Internal Medicine and Gastroenterology, Carol Davila University of Medicine and Pharmacy, 8 Eroii Sanitari Str., 050474 Bucharest, Romania; florentina.ionita-radu@umfcd.ro; 7 Department of Gastroenterology, “Dr. Carol Davila” Central Military Emergency University Hospital, 134 Calea Plevnei Str., 010242 Bucharest, Romania

**Keywords:** multiple sclerosis, thrombotic thrombocytopenic purpura, interferon beta-1a

## Abstract

Background: Secondary thrombotic thrombocytopenic purpura (TTP) due to interferon beta-1a intramuscular (im) treatment is an uncommon adverse effect with only a few cases in multiple sclerosis patients reported worldwide. TTP together with haemolytic uremic syndrome (HUS) are classic forms of thrombotic microangiopathy, characterized by small-vessel platelet micro-thrombi that manifest clinically in a similar manner. Most common signs and symptoms include bruises and ecchymosis, neurologic symptoms and renal impairment. Interferon beta-1a represents one of the first-line therapies for relapsing-remitting multiple sclerosis due to its accessibility and efficacy. Case presentation: A 36-year-old woman who was previously diagnosed with relapsing-remitting multiple sclerosis had received weekly intramuscular injections with beta-interferon-1a (Avonex 30 mcg). After 9 months of treatment, she presented bruises and ecchymosis on her limbs and torso, epistaxis, gingival bleeding aggravated within 48 h and a persistent headache that was non-responsive to common analgesics. Haematology tests revealed typical results for thrombotic microangiopathy, including severe thrombocytopenia (4000/mm^3^) and microangiopathic haemolytic anaemia with frequent schistocytes on the peripheral blood smear. Once the beta-interferon administration was ceased and upon the initiation of methylprednisolone, the symptoms remitted. Conclusions: In this case study, we portrayed the particular association between the remission phase of multiple sclerosis and the violent onset of interferon-induced thrombotic thrombocytopenic purpura.

## 1. Introduction

Multiple sclerosis (MS) is the most common inflammatory demyelinating disease of the central nervous system, characterized by myelin damage, axonal loss and blood−brain barrier disruption. Despite the foregoing data, the autoimmune mechanisms in MS are not entirely explained. However, the role played by certain inflammatory cytokines such as interferon-gamma, tumour necrosis factor-alpha and interleukins, upholds the use of disease-modifying therapies (DMT) for MS.

Beta interferons (IFN-β) represent the first class of DMTs approved for relapsing-remitting multiple sclerosis (RRMS), with an overall favourable safety profile. Frequent complications associated with IFN-β treatment include flu-like symptoms, headache, anorexia and impairment of the hematopoietic, cardiovascular and gastrointestinal systems [1].

Thrombotic microangiopathy (TMA) is a rare but life-threatening side effect of the intramuscular formulation of interferon beta-1a (Avonex 30 mcg), being previously described in the literature in two case reports [2]. A recent cumulative review has reported a relatively high incidence of TTP in MS patients treated with subcutaneous (sc) IFN β-1a (Rebif), at approximately 30% of the patients included in the study [3]. It has also been suggested that interferon-induced TMA is linked to a toxic dose-related mechanism [4]. The two most common forms of TMA are thrombotic thrombocytopenic purpura (TTP) and haemolytic uremic syndrome (HUS), both presenting similar clinical manifestations, with additional kidney dysfunction for the latter [5]. Systemic platelet aggregation in TTP often leads to organ ischemia, increased consumption of thrombocytes resulting in profound thrombocytopenia (with increased marrow megakaryocytes) and microangiopathic haemolytic anaemia [6].

Our case of a 36-year-old woman diagnosed with RRMS and secondary TTP induced by im IFN-βa (Avonex, Biogen Netherlands B.V.) treatment, illustrates the importance of careful monitoring of hematologic parameters when administrating beta-interferons.

## 2. Case Report

**Figure 1:**
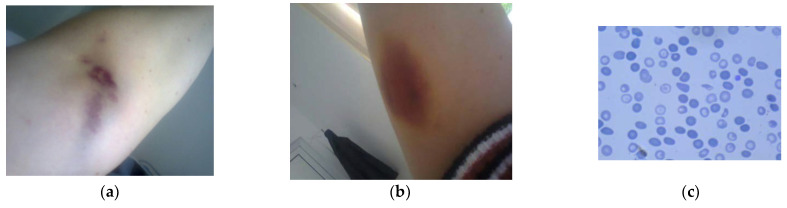
(**a**,**b**). The patient’s forearm presents multiple bruising and ecchymosis. (**c**). Peripheral blood smear in Giemsa coloration: frequent schistocytes.

We present the case of a 36-year-old right-handed woman who was diagnosed with RRMS, following the 2017 revised McDonald criteria, and promptly included in our national health program for MS patients [7]. Given the clinical history of our patient, who presented a single clinical exacerbation, and the MRI findings, which reported a single active lesion, we initiated the administration of beta-interferon-1a (Avonex) once per week. Following 9 months of weekly im injections, the patient was admitted after developing multiple bruises and ecchymosis on her limbs and torso, associated with epistaxis and gingival bleeding (Figure 1a,b). A few days before the onset of her symptomatology, the patient complained of a severe headache that was non-responsive to common analgesics (naproxen 220 mg).

During the interferon treatment, the patient’s blood pressure and blood tests were monitored every 3 months, including complete blood count (CBC), liver enzymes and renal function, with no abnormal results. Moreover, she has had no clinical relapse since the initiation of Avonex. The initial diagnosis screening revealed severe thrombocytopenia (4000/mm^3^), increased WBC, lymphocytopenia and microangiopathic haemolytic anaemia with frequent schistocytes on the peripheral blood smear (Figure 1b). A brain and cervical spine MRI showed no active demyelinating lesions compared to the moment of MS diagnosis.

Repeated blood tests revealed increased lactate dehydrogenase levels, normal liver enzymes and normal kidney function. Coagulation assay and fibrinogen were within range. Immunological tests (anti-dsDNA, anti-La/SS-B antibodies, IgM and IgG anti-phospholipid antibodies, complement levels), as well as infectious disease tests (hepatitis B virus, hepatitis C virus, human immunodeficiency virus) and cancer markers, were within range. To exclude other causes of purpura we tested for known coagulopathies, including the Von Willebrand Factor Antigen (Factor VIII: R), which rendered a normal value. Hepatosplenomegaly, abdominal or pelvic bleeding and pregnancy were excluded by performing ultrasound.

Given the progressive decrease in the thrombocytes count, the haematologist report strongly advised for platelet mass transfusion (PMT), hence the patient received 6 units of PMT. Upon the interruption of interferon administration and the initiation of high-dose intravenous methylprednisolone (1000 mg/day for 3 consecutive days) the patient had a complete remission of the symptoms and the normalization of the blood tests.

## 3. Discussion

Although MS and other autoimmune disorders, such as idiopathic thrombocytopenic purpura, systemic lupus erythematosus, autoimmune thyroiditis and myasthenia gravis, can co-exist, up till now there are no reported cases of TTP in naïve MS patients [1]. Subcutaneous administration of interferons is documented to have high causality concerning the TMA/TTP-HUS spectrum. Ben-Amor et al. described a causal association between sc IFN β-1a treatment and TMA/TTP-HUS in 67% of the included patients. However, intramuscular administration differs for the absorption rate and recommended dose; im IFNβ-1a 30 µg is administered once per week, compared to sc IFNβ-1a 44 µg at three times per week [8,9]. Published results from the phase 4, long-term observational PROOF study (NCT00292266) highlighted that sc IFNβ-1a (Rebif) is more immunogenic than the intramuscular alternative, which might offer insight regarding a possible pathophysiological mechanism [10]. Other case reports with similar findings have been published, nevertheless, without any consensus about officially registering TTP as an adverse effect of sc IFN β-1a [11,12]. However, the summary of the product characteristics of Avonex includes TMA/TTP-HUS as a rare undesirable effect and recommends the close monitoring of patients receiving this treatment [9].

The management of TTP requires fast decisions and timely recognition. Patients with acute TTP rapidly become clinically unstable and often present sudden and unpredictable deterioration. Left untreated, TTP is associated with extremely high mortality rates, in up to 90% of cases. Current guidelines recommend immediate initiation of plasma exchange (PEX), as it has proven to reduce mortality by up to 10–20% in TTP patients [13]. The next therapeutic approach is steroid administration, preferably intravenous methylprednisolone (1000 mg/day, 1–3 days), followed by particular treatment options depending on the co-existence of special conditions such as HIV-positive cases or cardiological symptomatology [14]. In our case, we focused on eliminating the presumable trigger of the pathology by interrupting the administration of IFN-βa. Plasma exchange was not available in our hospital at that moment, hence we opted for the second-in-line option of initiating steroid perfusion.

Regarding the platelet mass transfusion in TTP, the published data in recent literature is still controversial. While some of the studies militate for the contraindication of performing platelet transfusion in TTP patients due to the high thrombosis risk, there is evidence that platelet mass is beneficial in TTP, especially when the thrombocyte count is extremely low [15].

In what concerns the therapeutic approach regarding MS treatment, multiple options were considered and investigated to avoid further complications. It is important to remark the low-aggressive profile of our patient’s neurological condition, hence appropriate treatment escalation should be considered. Following careful literature revision, haematological advice and the patient’s preference for oral medication, we decided on teriflunomide (Aubagio). Thrombocytopenia due to immune phenomena is a reportedly rare adverse effect of teriflunomide, as multiple safety and tolerability studies have reported [16,17]. For the past three years, our patient had no documented clinical relapse of MS. Periodic monitorization of blood tests has not revealed abnormal values so far.

Given the sequence of events in our patient, TTP is highly suspected to have been acquired, given its prompt remission after ceasing the current MS medication.
